# Patient-derived high functional avidity TCR empowers diverse T cell subsets for tumour immunotherapy

**DOI:** 10.3389/fimmu.2026.1964649

**Published:** 2026-10-19

**Authors:** Fei Gao, Yanchun Peng, Ji-Li Chen, Mariana Pereira Pinho, Xuan Yao, Yi-Ling Chen, Uzi Gileadi, Adam Bates, Craig Waugh, Paul Sopp, Nikolaos I. Kanellakis, Najib M. Rahman, Ricardo A. Fernandes, Graham S. Ogg, Tao Dong

**Affiliations:** 1 CAMS Oxford Institute, Chinese Academy of Medical Sciences & Peking Union Medical College, University of Oxford, Oxford, United Kingdom; 2 MRC Translational Immune Discovery Unit (TIDU), MRC Weatherall Institute of Molecular Medicine (WIMM), Radcliffe Department of Medicine (RDM), University of Oxford, Oxford, United Kingdom; 3 Oxford Centre for Respiratory Medicine, Churchill Hospital, Oxford University Hospitals NHS Foundation Trust, Oxford, United Kingdom; 4 Oxford National Institute of Health Research (NIHR) Biomedical Research Centre, Oxford University Hospitals NHS Foundation Trust, Oxford, United Kingdom; 5 Laboratory of Pleural and Lung Cancer Translational Research, Nuffield Department of Medicine, University of Oxford, Oxford, United Kingdom

**Keywords:** adoptive cell therapy (ACT), T cell engineering, T cell receptor (TCR), TCR-T cell, tumour immunotherapy, tumour-reactive T cell

## Abstract

Cancer-testis antigens (CTAs) are attractive targets for tumour immunotherapy due to their restricted expression in human malignancies and broad population-level prevalence. However, naturally occurring CTA-reactive T cells are often exhausted or functionally compromised, limiting their direct therapeutic utility. Here, using a non-viral orthotopic TCR replacement system, we systematically evaluated two patient-derived HLA-A*02:01-restricted TCRs specific for the synovial sarcoma X breakpoint 2 (SSX2) epitope SSX2_41-49_ (KASEKIFYV). Although both TCRs conferred SSX2 specificity, one displayed markedly higher functional avidity and superior antigen responsiveness. Primary CD8^+^ T cells redirected with this high-avidity TCR showed CD8-independent recognition, enhanced tetramer binding, and stronger functional responses against SSX2-expressing tumour cells. Notably, the same TCR also redirected CD4^+^ T cells toward the MHC-I-restricted SSX2 epitope, inducing polyfunctional cytokine release and direct tumour cytotoxicity. Moreover, it remained highly functional when introduced into unconventional T cell subsets, including NKT and γδ T cells, thereby expanding the range of potential cellular platforms for TCR-based therapeutics. Together, these findings identify a naturally derived, high functional avidity SSX2-reactive TCR with broad activity across conventional and unconventional T cell subsets, supporting its further development for future TCR-T cell-based immunotherapies.

## 1 Introduction

Tumour antigens are immunogenic targets for the adaptive immune system (1), and T cells have attracted particular attention due to their antigen-specific recognition and capacity to eliminate cancerous cells (2). Numerous studies have demonstrated the crucial role of T cells in tumour immunosurveillance, particularly given that the presence of tumour-reactive T cells is associated with improved clinical outcomes across multiple cancers (3–5). Therefore, immunotherapy strategies aim to convert immune “cold” tumours into immune “hot” tumours, primarily by enhancing cytolytic T lymphocyte (CTL) infiltration and cytotoxicity against tumour antigen-expressing cells (6). Immune checkpoint inhibitors (ICIs), such as PD-1 and CTLA-4 antibodies, ameliorate T cell exhaustion but may cause immune-related adverse events due to broad, antigen-nonspecific T cell activation (7–9). Moreover, the reliance on pre-existing tumour-reactive T cells and the substantial non-responsive rates underscore the need for robust and precise immunotherapeutic approaches.

Genetically modified T cells, particularly chimeric antigen receptor (CAR)-T and TCR-T cells, offer a promising solution by generating a replenishable supply of tumour-reactive T cells through the exogenous introduction of tumour-reactive receptors into recipient T cells (10, 11). While CAR-T cell therapy has achieved remarkable success in B-cell malignancies (12, 13), its clinical application remains largely confined to haematological cancers and has shown limited efficacy against solid tumours, mainly owing to its reliance on cell-surface antigen recognition (14). In contrast, TCR-T cells exploit the natural ability of TCRs to recognize peptide-MHC complexes, enabling the precise targeting of both cell-surface and intracellular tumour antigens (15). The broader antigen-recognition capacity positions TCR-based therapies as an attractive strategy across a wide range of cancers (16).

CTAs represent a unique class of tumour-associated antigens (TAAs) and are considered ideal immunotherapy targets due to their tumour-restricted expression and the absence from most normal tissues (17). CTA-targeting cellular therapies, such as those targeting NY-ESO-1 and MAGE-A4 have demonstrated remarkable success in recent clinical applications (1). To broaden therapeutic reach, we focused on the synovial sarcoma X breakpoint 2 (SSX2) antigen, a frequently occurring CTA across multiple human cancers, including acute myeloid leukaemia (AML), synovial sarcoma, melanoma, prostate cancer, non-small cell lung cancer (NSCLC), and malignant pleural mesothelioma (MPM) (18–20). Therefore, identification of a functionally competent SSX2-targeting TCR would enable the development of “off-the-shelf” therapeutics to benefit large patient cohorts.

Unconventional T cells, such as γδ T cells, MAIT cells, and NKT cells, are gaining particular attention as promising immunotherapeutic candidates, whereas conventional therapies have predominantly focused on αβ T cells due to their abundance in peripheral blood (21). These unconventional T cells exhibit potent innate-like cytotoxicity, rapid effector responses, and a remarkable capacity to infiltrate immunosuppressive tumour microenvironments (TME) (21, 22). Moreover, the presence of unconventional T cells in various tumours positively correlates with improved patient survival, underscoring their intrinsic role in tumour control (23). Therefore, leveraging unconventional T cells may enable immunotherapy to overcome key limitations of conventional approaches, offering a versatile strategy against resistant or heterogeneous cancers (24).

In this study, we systematically characterised patient-derived SSX2-specific TCRs using an orthotopic TCR replacement system across multiple T cell subsets. We identified a naturally occurred, high functional avidity TCR with CD8-independent functionality that also exhibited superior tumour-suppressing activity. Remarkably, this TCR demonstrated a unique ability to enable CD4^+^ T cell activation through the MHC-I-restricted SSX2_41–49_ epitope, conferring both helper functions and direct cytotoxicity for tumour control. Moreover, this TCR effectively redirects unconventional T cells with tumour specificity, further broadening its therapeutic applicability. These findings indicate that this SSX2-reactive TCR combines superior intrinsic functionality with the capacity to mediate effective tumour control across diverse T cell subsets. Collectively, our study identifies a compelling and versatile TCR candidate with strong potential for the development of future TCR-based tumour immunotherapy strategies.

## 2 Materials and methods

### 2.1 Study ethics and human samples

Cancer patients were recruited by the Oxford Pleural Unit (Oxford University Hospitals NHS Trust, 2019-2023). Samples were collected under Oxford Radcliffe Pleural Biobank ethical approval (19/SC/0173, South Central - Oxford C Research Ethics Committee). The study was approved by the Oxford Radcliffe Biobank Tissue Access Committee (19/A022). Peripheral blood mononuclear cells (PBMCs) were obtained from healthy donors under NHS REC 21/PR/1155 approved by NHS South Central-Oxford C Research Ethics Committee. All participants provided written informed consent.

### 2.2 PBMC and T cell isolation

PBMCs were isolated using density gradient centrifugation as previously described (25). Briefly, 30 mL blood was added to the 50 mL Falcon tube with 15 mL Lymphoprep (Stem Cell Technologies) and centrifuged at 2,000 rpm for 20 mins (accelerate 2, break 0) at room temperature (RT). The lymphocytes were then transferred into a new tube and washed with RPMI 1640 for subsequent cell counting.

Primary CD8^+^ and CD4^+^ T cells were isolated from PBMCs using the Miltenyi Biotec’s magnetic selection kits (Miltenyi, Germany). Specifically, CD4^+^ T cells were isolated by the CD4^+^ T cell positive selection kit and CD8^+^ T cells were isolated from the rest of the unlabelled cells using a CD8 negative selection kit following the manufacturer’s instructions. Isolated T cells were plated at 2 × 10^6^ cells/mL in a 24 or 48-well culture plate and were cultured in H10 medium (RPMI 1640 with 10% human AB serum) in the presence of 200 IU recombinant human IL-2.

### 2.3 TCR sequencing from SSX2_41-49_-specific CD8^+^ T cell clones

TCR sequencing of the SSX2_41-49_-reactive T cell clones was performed as previously described (26). Briefly, mRNA from T cell clones was extracted using the RNeasy Mini Kit (Qiagen) and cDNA was synthesized with the SMARTer RACE cDNA Amplification kit (Takara) according to the manufacturer’s instructions. The CDR3 region of TCR alpha and beta chains was amplified with the PCR Advantage Kit (Takara) and the PCR product was purified with QIAquick PCR Purification Kit (Qiagen) prior to sequencing.

### 2.4 Gene modified T cell design and primary T cell engineering

Primary T cell engineering was performed as previously described with modifications (27). Briefly, CRISPR RNPs were generated by annealing each guide RNA (sgRNA, Integrated DNA Technologies, USA) with Alt-R S.p. Cas9 Nuclease V3 protein (Integrated DNA Technologies) at 3:1 molar ratio at room temperature for 15–20 mins prior to electroporation. Murinized TCR template for HDR repair was designed as “5’ homologous arm-P2A-TCRβ-T2A-TCRα-bGHpA tail-3’ homologous arm” (28). gRNA sequence for *TRAC* was 5′-AGAGTCTCTCAGCTGGTACA-3′; 5′-GGAGAATGACGAGTGGACCC-3′ for both *TRBC1* and *TRBC2*; 5’-CATGACAAAAACGGATGGTT-3’ for *TRDC*. Exogenously designed TCRs were codon optimized to facilitate expression and the full-length DNA donor templates were synthesized by GeneArt (Life Technologies).

T cells were activated by ImmunoCult Human CD3/CD28 T cell Activator (Stem Cell Technologies) 2 days prior to electroporation following the manufacturer’s instructions. Upon activation, 1 × 10^6^ T cells were washed and resuspended in 20 μl P3 Primary Cell Nucleofector Solution (Lonza) and electroporated (pulse code EO-115) with previously generated CRISPR RNPs in the presence of the HDR repair template using a 4D Nucleofector X unit (Lonza). Cells were then transferred to a 48-well plate with pre-warmed H10 culture medium after electroporation and kept in culture in 5% CO_2_ at 37 °C.

FACS sorted T cells were cultured using feeder cells (irradiated PBMCs) in the presence of 50 μg/mL PHA and 200 IU recombinant human IL-2. Culture medium was changed at every two-day interval. T cells were used for subsequent functional evaluations between day 12 and 16 post-feeder stimulation.

### 2.5 SSX2 antigen expressing target cell line generation

Human *SSX2* (isoform a) gene (NM_003147) was used for SSX2 antigen expression. For the vaccinia virus infection assay, Epstein-Barr virus (EBV)-transformed immortal B cells (BCLs) were infected with SSX2 or SSX4 antigen engineered vaccinia virus at an MOI of 3 for 2 hours at 37 °C. Cells were then washed with R10 to remove vaccinia virus residues and recovered overnight for antigen expression. For lentivirus transduced cell lines, the *SSX2* gene sequence was cloned into an eGFP co-expressing lentiviral vector. The empty vector (EV) control and the SSX2-expressing plasmids were co-transfected with pMD2.G and psPAX2 packaging plasmids into HEK293T cells with PEIpro (Polyplus). Supernatants were collected at 48 and 72 h post-transfection and were then added to HCT 116 and MESO 174 cells.

### 2.6 FACS staining and flow-based cell sorting

Cells were first stained with Zombie Aqua™ Fixable Viability Kit (BioLegend) for live/dead cell staining at 4 °C in the dark for 10 mins. Cells were then washed with FACS washing buffer (PBS with 0.5% BSA and 2 mM EDTA) and stained with subsequent antibodies for surface staining at 4 °C in the dark for 20 mins: hTCRαβ (IP26, BioLegend), mTRBC (H57-597, BioLegend), hTCR γ/δ (B1, BioLegend), CD4 (SK3, BioLegend), CD8 (SK1, BioLegend). The HLA-A*02:01/SSX2_41-49_ (KV9) tetramer was generated in-house and was stained for 20 mins at 37 °C in the dark. All samples were acquired on Attune NxT Flow Cytometer (ThermoFisher) and flow-based cell sorting was performed on the FACSAria platform (BD Biosciences).

### 2.7 Intracellular cytokine staining

Intracellular cytokine staining (ICS) was performed as previously described (29). Briefly, primary T cells were co-cultured with peptide-pulsed T2 cells, vaccinia virus infected BCLs or cancer cell lines with Golgi plug (BD Biosciences), Golgi stop (BD Biosciences) and CD107a (H4A3, BD Biosciences) antibody at 37 °C for 4 hours. After incubation, cells were stained with Live/Dead zombie dye and then fixed and permeabilized with Cytofix/Cytoperm (BD Biosciences) and stained intracellularly for IFN-γ (B27, BD Biosciences), TNF-α (MAb11, eBiosciences), IL-2 (MQ1-17H12, BioLegend) and MIP-1β (D21-1351, BD Biosciences). Samples were then acquired by the Attune NxT Flow Cytometer (ThermoFisher).

### 2.8 FACS based T cell cytotoxicity assay

FACS based T cell cytotoxicity assay was performed as previously described (30). Briefly, target T2 cells were labelled with Cell-TraceViolet (Invitrogen) before being pulsed with peptide and then co-cultured with TCR-T cells at 1:1 ratio. 24 hours after co-culture, Far Red (Invitrogen) labelled T2 cells were added into each well as reference cells for FACS analysis. Cells were then harvested and were read on the Attune NxT Flow Cytometer (ThermoFisher). The percentage of T cell-mediated killing for each well was calculated with the following equation: % cytotoxicity = 100 − ((% target cell/% reference cell)/(% unkilled target cell control/% reference cell) × 100). The % target cell control was determined by culturing T2 cells in the absence of effector T cells.

### 2.9 Real-time measurement of T cell cytotoxicity

Target cells were co-cultured with TCR-T cells in a flat bottom 96-well plate (Corning) at 1:1 ratio. The plate was then placed into the IncuCyte imaging system (Sartorius) to track the fluorescence intensity for each well at every 2-hour intervals for up to 72 hours. For T2 cells, CellTox (Promega) was added in the culture medium and the killing index was calculated by normalizing the mean fluorescent intensity at each time point to the basal level mean fluorescent intensity. For SSX2 over-expressed tumour cell lines, relative tumour growth was measured by normalizing GFP intensity at each time point to the basal level GFP intensity.

### 2.10 Statistical analysis

TCGA and GTEx data were analysed using the RStudio v1.4.1106. FACS data were analyzed by using FlowJo V.10.10.0. DNA sequencing data were analysed with SnapGene v.4.3.7. All graphs were generated and statistical analyses were performed by using GraphPad Prism v.9 software. All statistical details and numbers of repeats were stated in the corresponding figure legends. Statistically significant differences between two groups were assessed by using Student’s *t*-test. One-way or two-way ANOVA, followed by Tukey’s *post hoc* multiple comparison test was performed to compare two or more groups. Unless stated otherwise, data were shown as mean ± SD. All tests were two-tailed, and statistical differences were set as *P < 0.05; **P < 0.01; ***P < 0.001, ****P < 0.0001, n.s. as not significant.

## 3 Results

### 3.1 SSX2-specific TCRs exhibit distinct functional avidities

While CTAs provide promising therapeutic targets for tumour immunotherapy, naturally occurring high functional avidity T cells with specific recognition of these antigens are rare. Using CTA peptide pool reactivity enrichment, followed by tetramer based single cell sorting and *in vitro* T cell expansion, two HLA-A*02:01-restricted SSX2_41-49_ (KASEKIFYV) reactive CD8^+^ T cell clones with different TCR sequences were isolated from either peripheral blood (PB T cell clone) or malignant pleural effusion (MPE T cell clone) from cancer patients (26) (**Figure 1A**). Initial functional evaluation of these two TCRs using lentivirus-transduced Jurkat cells showed the MPE-derived TCR had greater functional potency than the PB-derived TCR (~20-fold functional difference) (**Supplementary Figure 1**). We therefore named these two TCRs the high and low functional avidity TCRs.

**Figure 1:**
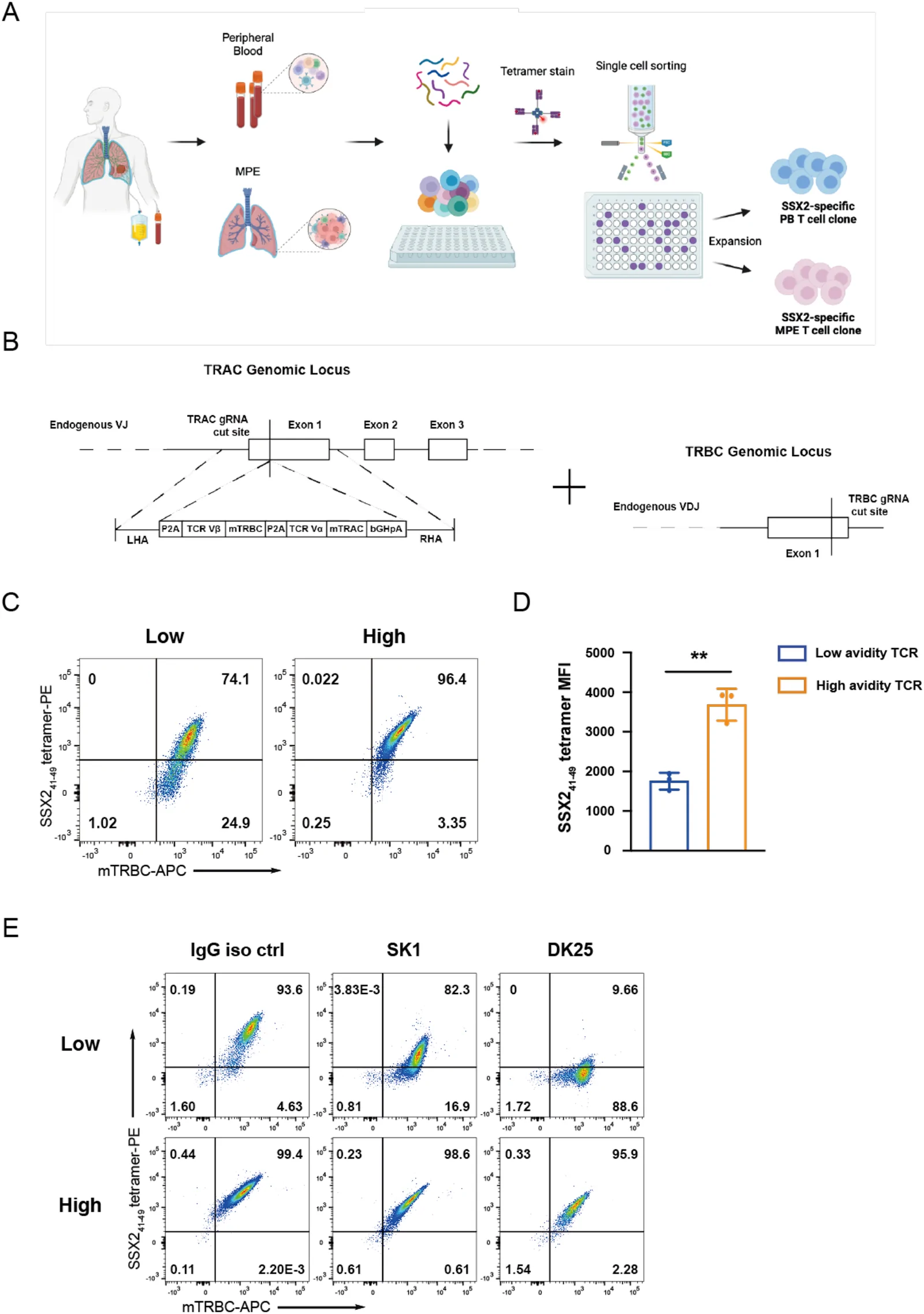
TCRs of the same specificity but different functional avidity. Characterising TCRs of SSX2 specificity but different functional avidity using an orthotopic TCR replacement system. **(A)** Schematic overview of SSX2-reactive T cell clones isolated from either PBMCs or malignant pleural effusions (MPE) of cancer patients. T cells were first stimulated by SSX2 peptide pool to enrich SSX2-reactive T cells, then the SSX2-reactive single T cell clones were generated by using the HLA-A*02:01/SSX2_41–49_ tetramer staining followed by single cell sorting and *in vitro* T cell expansion. Figure was generated by using BioRender at https://www.biorender.com **(B)** Schematic overview of generating SSX2-reactive TCR-T cells. Murinized exogenous SSX2-reactive TCRs were inserted into the endogenous *TRAC* gene locus, together with disruption of the endogenous *TRBC* gene expression. **(C)** Representative flow cytometry staining of HLA-A*02:01/SSX2_41–49_ tetramer and mTRBC on *in vitro* expanded SSX2-reactive CD8^+^ TCR-T cell surface. **(D)** Flow cytometry analysis of mean fluorescent intensity (MFI) of HLA-A*02:01/SSX2_41–49_ tetramer binding (n=3) on SSX2-reactive CD8^+^ TCR-T cell surface. **(E)** Representative flow cytometry staining of HLA-A*02:01/SSX2_41–49_ tetramer and mTRBC expression on CD8^+^ TCR-T cell surface after anti-CD8 antibody or IgG isotype blockade. Data are presented as mean ± SD. Unpaired Student’s *t*-test **(D)** was performed, and two-tailed P values were calculated for comparison. *P < 0.05; **P < 0.01; ***P < 0.001.

To systematically characterize these TCRs, we adopted the orthotopic TCR replacement system, as this strategy avoids the interference of the endogenous TCRs and provides a versatile platform to compare different exogenous TCR functions in parallel (28). Using CRISPR-mediated homology directed repair (HDR), we inserted the exogenous SSX2 TCRs with murinized constant regions into the *TRAC* gene locus while disrupting endogenous *TRBC* gene expression to prevent TCR mispairing (**Figure 1B**). In this way, we generated TCR-T cells from primary CD8^+^ T cells, and these cells showed robust surface TCR expression and antigen specific reactivity, as confirmed by surface murine TCR antibody (mTRBC) and HLA-A*02:01/SSX2_41–49_ tetramer staining (**Figure 1C**). Notably, the high functional avidity TCR demonstrated significantly higher tetramer binding potential compared to the low avidity counterpart (**Figure 1D**). Additionally, the subsequent CD8 antibody blocking assay showed the tetramer binding of the low avidity TCR was completely abrogated by the anti-CD8 antibody clone DK25 and partially blocked by the clone SK1. In contrast, the tetramer binding of the high avidity TCR was only slightly affected under the same conditions (**Figure 1E**). Together, these findings indicate the CD8-independent nature of the high functional avidity SSX2-reactive TCR, underscoring its translational potential for broad therapeutic applications.

### 3.2 Functional evaluation of SSX2-reactive TCRs

To functionally characterise SSX2_41-49_-reactive TCRs on primary T cells, we first co-cultured TCR-T cells with SSX2_41–49_ peptide titrated T2 cells and performed intracellular cytokine staining (ICS) to assess cytokine release upon activation. IFN-γ and TNF-α production confirmed that the high avidity TCR exhibited enhanced sensitivity towards tumour antigens, as indicated by its remarkably low EC_50_ level (**Figures 2A, B**). As the peptide-titration response reached a plateau at 1000 ng/mL, this concentration was selected as a saturating positive-control condition for subsequent single-dose validation assays. Moreover, TCR-T cell responses to JEG-3 cells further confirmed the HLA-A*02:01-restricted recognition of the SSX2-reactive TCR, as SSX2_41–49_ peptide-induced activation was only observed in HLA-A*02:01^+^ JEG-3 cells but not in parental HLA-A*02:01 negative JEG-3 cells (**Supplementary Figure 2**). Next, we evaluated the recognition of naturally processed and presented SSX2 antigen by co-culturing TCR-T cells with B cells infected with vaccinia virus engineered to express the SSX2 antigen. ICS results confirmed antigen specific recognition by both TCRs, with the high avidity TCR showing robust reactivity, whereas a weak response was seen with the low avidity TCR (**Figure 2C**). Lastly, *in vitro* killing assays were performed by co-culturing TCR-T cells with peptide titrated T2 cells, and the high avidity TCR displayed enhanced cytotoxicity compared to the low avidity TCR (**Figures 2D–F**). The remarkably lower EC_50_ level and greater cytotoxicity were observed for the high avidity TCR at very low peptide concentrations (0.1 ng/mL and 1 ng/mL), suggesting its functional potency at extremely low tumour antigen loads (**Figures 2D, E**). Additionally, we monitored real-time killing kinetics using the IncuCyte system at very low peptide concentrations. The high avidity TCR started to suppress target cell growth at 0.1 ng/mL SSX2_41–49_ peptide concentration and exhibited robust cytotoxicity at 1 ng/mL SSX2_41–49_ peptide concentration (**Figure 2F**). In contrast, the low avidity TCR showed minimal killing under the same conditions (**Figure 2F**). Collectively, these results indicate that the SSX2-reactive high avidity TCR is functionally more potent than its low avidity counterpart. To further evaluate the translational specificity of the SSX2-reactive TCR, we performed a preliminary *in silico* screen for human self-peptides with sequence similarity to the SSX2_41–49_ epitope (KASEKIFYV). Candidate 9-mer peptides with ≤3 amino-acid mismatches were further assessed for predicted HLA-A*02:01 binding/presentation and source-protein expression in normal tissues. This analysis identified a limited set of candidate self-peptides for subsequent experimental prioritization (**Supplementary Table 1**).

**Figure 2:**
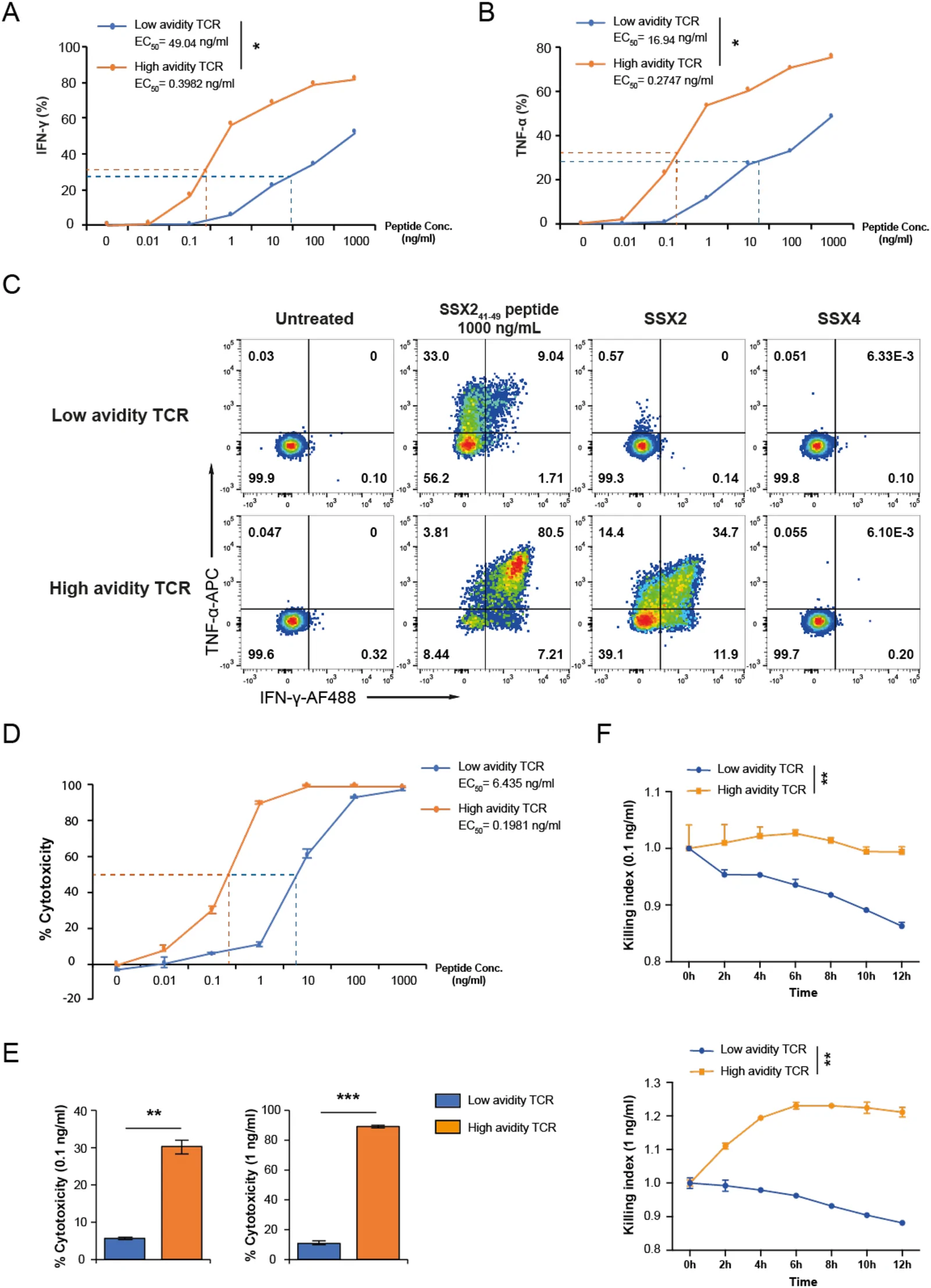
The high avidity TCR is more potent than its low avidity counterpart. Functional comparison of SSX2-reactive TCRs by using Homology Directed Repair (HDR) engineered CD8^+^ TCR-T cells. **(A, B)** Flow cytometry analysis and EC_50_ calculation of intracellular IFN-γ **(A)** and TNF-α **(B)** release after SSX2-reactive TCR-T cells co-cultured with SSX2_41–49_ peptide titrated T2 cells. Peptide concentration: ng/mL. **(C)** Representative flow cytometry staining of intracellular IFN-γ and TNF-α release after SSX2-reactive TCR-T cells co-cultured with SSX2 or SSX4 antigen expressing vaccinia virus-infected or HLA-A*02:01 positive B cells pulsed with 1000 ng/mL SSX2_41–49_ peptide. **(D, E)** Cytotoxicity of low and high avidity SSX2-reactive TCR-T cells against SSX2_41–49_ peptide-pulsed T2 cells. Flow cytometry analysis and EC_50_ calculation of killing capacity between the low and high avidity TCRs **(D)** and killing capacity of SSX2-reactive TCR-T cells towards SSX2_41–49_ peptide-pulsed T2 cells at 0.1 ng/mL and 1 ng/mL peptide concentrations **(E)**. Data shown in **(D)** and **(E)** are from one representative donor of three technical replicate wells. Similar results were observed using TCR-T cells generated from 3 independent donors. Peptide concentration: ng/mL. **(F)** IncuCyte analysis of killing index of SSX2-reactive TCR-T cells towards SSX2_41–49_ peptide-pulsed T2 cells at 0.1 ng/mL and 1 ng/mL peptide concentrations at different time points. Data are presented as mean ± SD. Paired Student’s *t*-test **(A, B, F)** and unpaired Student’s *t*-test **(E)** were performed, two-tailed P values were calculated for comparison. *P < 0.05; **P < 0.01; ***P < 0.001.

### 3.3 Robust anti-tumour responses of TCR-T cells against SSX2-expressing tumour cell lines

As the SSX2-reactive high functional avidity TCR demonstrated potent function toward SSX2_41–49_ peptide loaded T2 cells and SSX2 antigen expressing B cells, we then sought to investigate its responsiveness to SSX2-expressing tumour cell lines. Initially, we established SSX2 antigen-expressing tumour cell lines by using HCT 116 and MESO 174 tumour cell lines, as these cell lines naturally express HLA-A*02:01 on the cell surface (**Supplementary Figure 3A**). These tumour cell lines were transduced with SSX2-GFP and GFP empty vector (EV) lentivirus, and GFP positive tumour cells were then sorted and expanded as target cells (**Supplementary Figure 4**). Co-culturing TCR-T cells with GFP expressing tumour cell lines showed that the high functional avidity TCR elicited a robust response in an SSX2 antigen-dependent manner (**Figure 3A**) and superior efficacy in suppressing tumour cell growth compared with its low avidity counterpart (**Figures 3B, C**). Notably, comparable levels of exhaustion markers were induced by both TCR-T cells post-killing (**Supplementary Figure 5**), suggesting that the enhanced functionality of the high functional avidity TCR did not come at the cost of increased T cell exhaustion.

**Figure 3:**
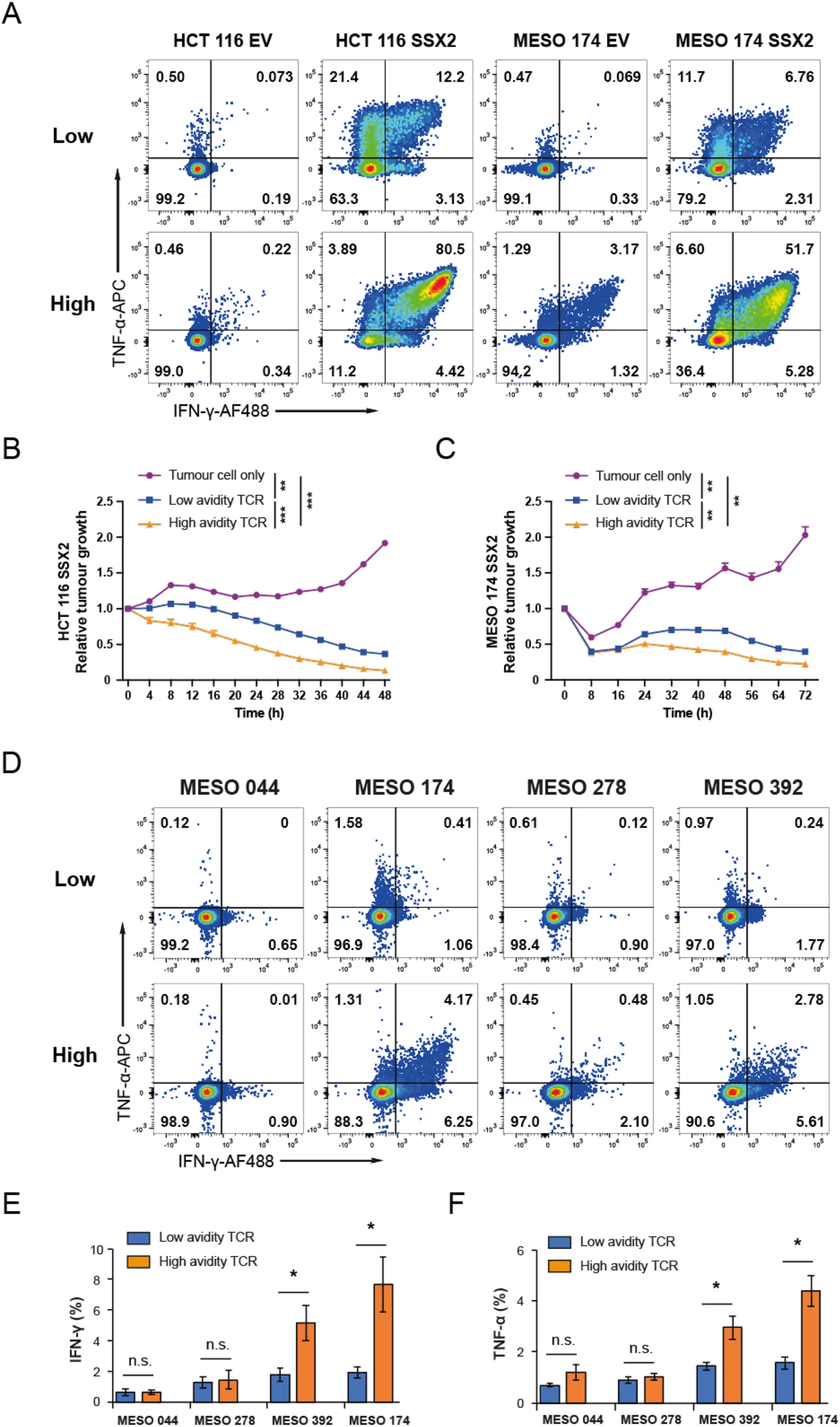
The high avidity TCR showed better tumour control than the low avidity TCR. Functional evaluation of SSX2-reactive TCR-T cells towards different tumour cell lines. **(A)** Representative flow cytometry staining of intracellular IFN-γ and TNF-α release after SSX2-reactive TCR-T cells co-cultured with GFP empty vector **(EV)** or SSX2-GFP (SSX2) lentivirus-transduced HCT 116 and MESO 174 tumour cell lines. **(B, C)** IncuCyte screening of SSX2-overexpressing HCT 116 **(B)** and MESO 174 **(C)** tumour cell line growth with or without co-culturing with SSX2-reactive TCR-T cells for 48 or 72h. **(D)** Representative flow cytometry staining of intracellular IFN-γ and TNF-α release after SSX2-reactive TCR-T cells co-cultured with mesothelioma patient-derived SSX2-expressing tumour cell lines. **(E, F)** Flow cytometry analysis of intracellular IFN-γ **(E)** and TNF-α **(F)** release of SSX2-reactive TCR-T cells towards different mesothelioma patient-derived tumour cell lines (n=3). Data were from one representative donor of three technical replicate wells and were presented as mean ± SD. One-way ANOVA with Tukey’s *post hoc* multiple comparison test **(B, C)** and unpaired Student’s *t*-test **(E, F)** were performed, two-tailed P values were calculated for comparison. *P < 0.05; **P < 0.01; ***P < 0.001, n.s., not significant.

Additionally, the TCR-T cell response towards the MESO 174 EV control was consistent with the previous report showing that the patient-derived SSX2-reactive T cell clone had a natural response against *SSX2*-expressing MESO cell lines (18). To confirm natural tumour antigen recognition, we evaluated TCR-T cell responsiveness against MESO cell lines with endogenous *SSX2* expression (**Supplementary Figure 3B**). The ICS results showed that except for MESO 044 cell line, which had the lowest SSX2 expression and downregulated HLA-A*02:01 on the tumour cell surface (**Supplementary Figures 3A, B**), both low and high avidity TCR-T cells responded to MESO cell lines (**Figure 3D**). The high functional avidity TCR showed significantly stronger reactivity towards MESO 174 and MESO 392 cell lines compared with the low avidity counterpart (**Figures 3E, F**). Analysis of the TCGA and GTEx databases also confirmed *SSX2* gene expression across multiple cancers of diverse tissue origins when compared with healthy and paratumour tissues (**Supplementary Figure 6**), indicating its potential applications for a broad spectrum of *SSX2*-expressing human malignancies. Collectively, these findings demonstrate the high sensitivity and functionality of the SSX2-reactive high functional avidity TCR for tumour immunotherapies.

### 3.4 High functional avidity TCR redirects CD4^+^ T cells through the MHC-I-restricted epitope

Having seen the distinct characteristics of these two TCRs in CD8^+^ T cells, we then sought to investigate their functionality by using CD4^+^ T cells, given the crucial role of CD4^+^ T cells in orchestrating tumour control (31). Similarly, we generated HDR TCR-T cells from PBMC-derived CD4^+^ T cells. TCR engineered CD4^+^ TCR-T cells confirmed the CD8-independence of the high avidity TCR, whereas the low avidity TCR completely lost tetramer binding potential in the absence of CD8 (**Figure 4A**). Subsequent co-culture assays with SSX2_41–49_ peptide titrated T2 cells showed that CD4^+^ T cells redirected with the high avidity TCR mounted potent polyfunctional cytokine release, including IFN-γ, TNF-α, CD107a, MIP-1β and IL-2, whereas those CD4^+^ T cells redirected with the low avidity TCR showed minimal activation even at the highest peptide concentration tested (**Figure 4B**). Consistent with these observations, CD4^+^ TCR-T cells co-cultured with vaccinia virus-infected B cell lines showed SSX2 antigen-specific activation in HLA-A*02:01 positive BCLs but not HLA-A*02:01 negative BCLs, further supporting HLA-A*02:01-restricted recognition and confirming the robust reactivity conferred by the high functional avidity TCR (**Supplementary Figure 7**).

**Figure 4:**
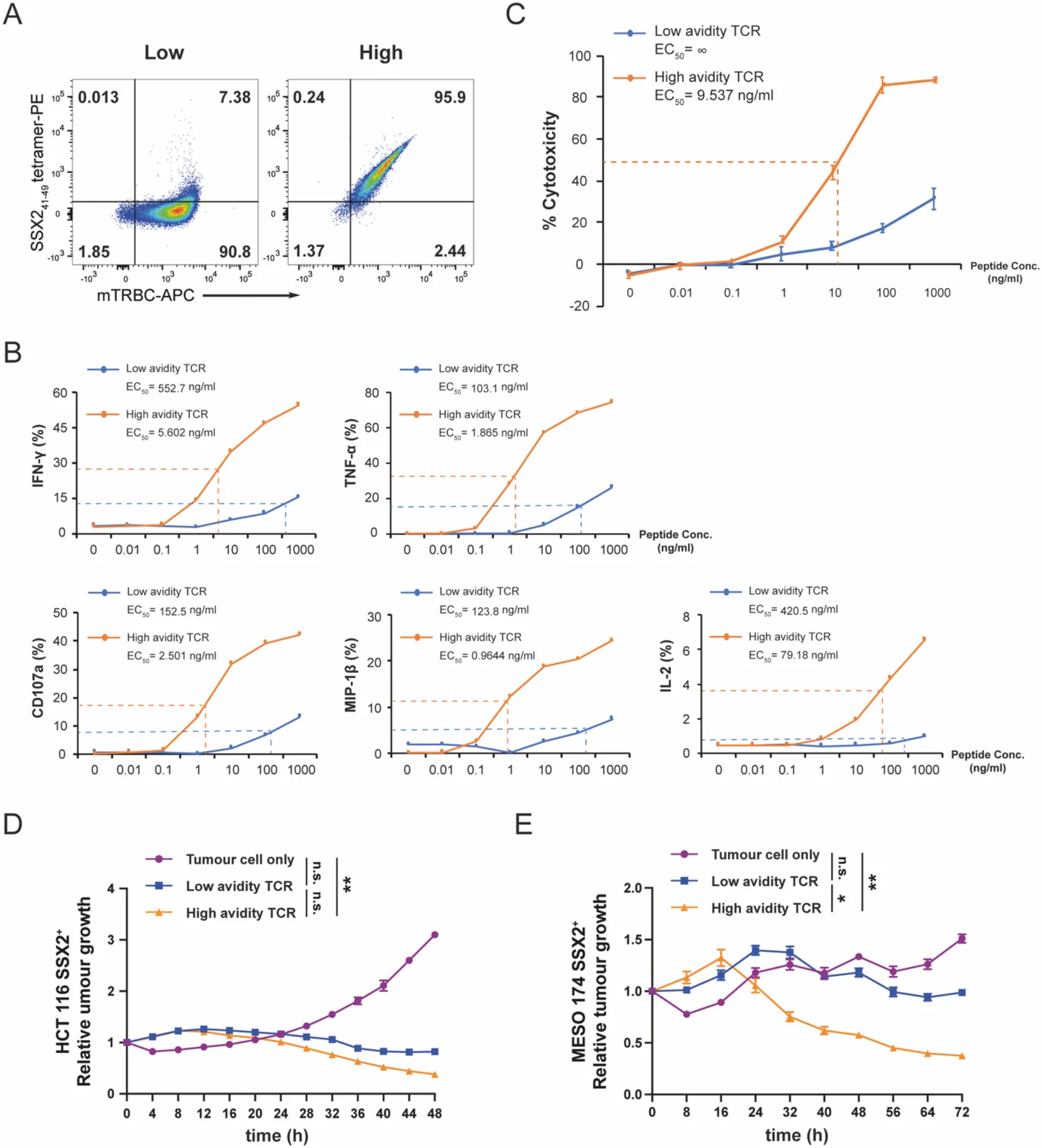
High avidity TCR triggers anti-tumour CD4^+^ T cell responses. Functional comparison of SSX2-reactive TCRs on CD4^+^ T cells. **(A)** Representative flow cytometry staining of HLA-A*02:01/SSX2_41–49_ tetramer and mTRBC on SSX2-reactive CD4^+^ TCR-T cell surface. **(B)** Flow cytometry analysis and EC_50_ calculation of intracellular IFN-γ, TNF-α, CD107a, MIP-1β and IL-2 release after TCR-T cells co-cultured with SSX2_41–49_ peptide titrated T2 cells. Peptide concentration: ng/mL. **(C)** Flow cytometry analysis and EC_50_ calculation of killing potential between SSX2-reactive low and high avidity TCRs towards SSX2_41–49_ peptide-pulsed T2 cells. Peptide concentration: ng/mL. **(D, E)** IncuCyte screening of SSX2-overexpressing HCT 116 **(D)** and MESO 174 **(E)** tumour cell line growth with or without co-culture with CD4^+^ TCR-T cells for 48 or 72h. Data are presented as mean ± SD. One-way ANOVA with Tukey’s *post hoc* multiple comparison test was performed **(D, E)** and adjusted P values were calculated for comparison. *P < 0.05; **P < 0.01; ***P < 0.001, n.s., not significant.

To further determine whether this activation translated into direct cytotoxic function, cytotoxicity assays demonstrated that CD4^+^ T cells redirected with the high avidity TCR effectively eliminated target cells upon activation (**Figure 4C**). Significant T2 cell elimination was observed at peptide concentrations above 10 ng/mL, whereas CD4^+^ T cells redirected with the low avidity TCR exhibited limited cytotoxicity under the same conditions (**Supplementary Figures 8A, B**). Furthermore, high functional avidity TCR redirected CD4^+^ TCR-T cells directly suppressed *in vitro* tumour cell growth, while those engineered with the low avidity TCR showed weaker anti-tumour activity (**Figures 4D, E**). Collectively, these findings further confirm the CD8-independent activity of the SSX2-reactive high avidity TCR and demonstrate its capacity to activate CD4^+^ T cells through an MHC-I restricted tumour epitope. Upon activation, these CD4^+^ TCR-T cells not only mediate direct tumour cell killing but also release a broad range of cytokines, thereby potentially enhancing tumour control.

### 3.5 Identification of unconventional T cells for tumour adoptive cell therapy

Given the potent anti-tumour function of the SSX2-reactive CD8-independent TCR, we explored its application in redirecting alternative immune cell subsets as recipients for transgenic TCR expression, aiming at leveraging their inherent characteristics for tumour control. Therefore, we FACS-sorted unconventional T cells, including NKT cells, MAIT cells and γδ T cells according to their lineage markers and expanded them before engineering (**Supplementary Figures 9A, B**). To extend non-viral HDR engineering to γδ T cells, we custom designed a *TRDC* targeting TCR replacement system, as this gene is conservatively expressed among γδ T cells and therefore could be utilized as a target for HDR-mediated gene delivery (**Supplementary Figure 10**). Following the same engineering strategy used for conventional αβ T cells, we generated unconventional TCR-T cells redirected by the SSX2-reactive high functional avidity TCR. Exogenous TCR expression was confirmed across all recipient cells via FACS staining for the murinized TCR marker (**Figure 5A**).

**Figure 5:**
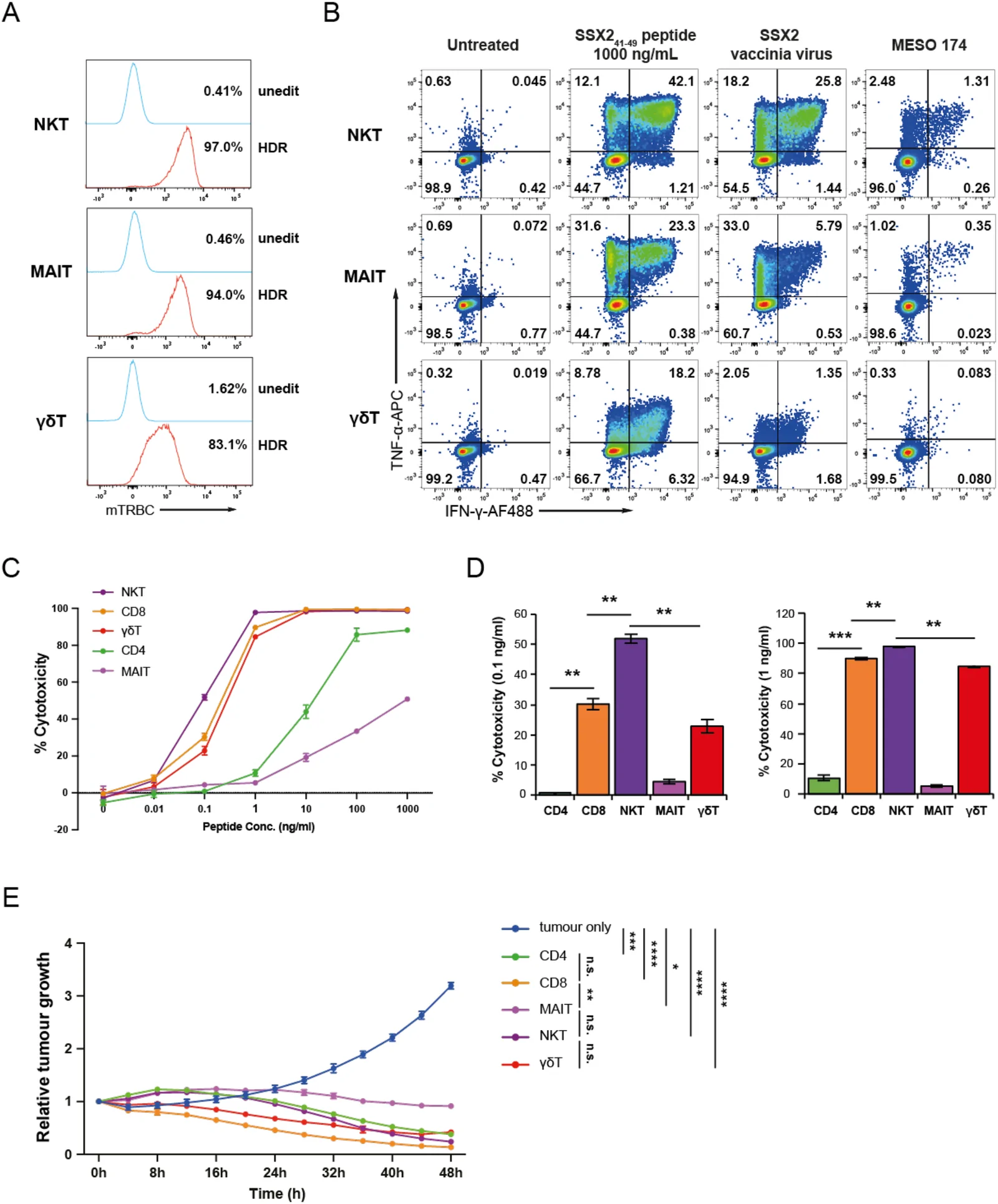
Identification of alternative T cell subsets for adoptive cell therapy. Unconventional T cells were redirected using the high functional avidity SSX2-reactive TCR. **(A)** FACS staining of mTRBC expression on unedited or SSX2-reactive TCR redirected unconventional T cell surface after editing and *in vitro* expansion. **(B)** Representative flow cytometry staining of intracellular IFN-γ and TNF-α release after unconventional TCR-T cells co-cultured with SSX2_41–49_ peptide (1000 ng/mL) pulsed T2 cells, SSX2 vaccinia virus infected B cells or MESO 174 tumour cell line. **(C)** Flow cytometry analysis of killing potential between SSX2-reactive TCR redirected conventional αβ T cells and unconventional T cells towards SSX2_41–49_ peptide titrated T2 cells. **(D)** Flow cytometry analysis of T cell killing potential comparison among different T cell subsets towards SSX2_41–49_ peptide-pulsed T2 cells at 0.1 ng/mL and 1 ng/mL peptide concentrations from **(C)**. **(E)** IncuCyte screening of SSX2 antigen expressing HCT 116 tumour cell growth 48 hours after co-culture with SSX2 high functional avidity TCR expressing conventional αβ TCR-T cells and unconventional TCR-T cells. Data are presented as mean ± SD and the one-way ANOVA with Tukey’s *post hoc* multiple comparison test was performed and adjusted P values were calculated for comparison **(D, E)**. *P < 0.05; **P < 0.01; ***P < 0.001, ****P < 0.0001, n.s., not significant.

The antigen specificity and TCR-T cell functionality of these unconventional T cells were confirmed with three different targeting approaches: peptide-pulsed T2 cells, HLA matched B cells infected with vaccinia virus engineered to express SSX2 antigen, and SSX2 antigen expressing tumour cell lines. Intracellular cytokine staining confirmed target-specific reactivity across all engineered T cells, although the cytokine profiles and response magnitude varied among different T cell types upon activation (**Figure 5B**, **Supplementary Figure 11**). To uniformly evaluate T cell effector function beyond cytokine secretion, we performed a cytotoxicity assay by using all T cell subsets engineered with the same TCR. T cell cytotoxicity assays revealed that compared with CD8^+^ T cells, both NKT and γδ T cells exhibited potent killing activity, which remained robust even at very low peptide concentrations. In contrast, MAIT cells and CD4^+^ T cells were less efficient in target cell elimination under the same conditions (**Figures 5C, D**). Moreover, all TCR-T cells suppressed tumour growth, although MAIT cells were less efficient than cytotoxic CD8^+^ T cells (**Figure 5E**). Taken together, these findings not only establish the feasibility of redirecting unconventional T cells with tumour specificity using a CD8-independent SSX2-reactive TCR, but also identify NKT and γδ T cells as promising candidates for next-generation T cell-based immunotherapies.

## 4 Discussion

The adoptive transfer of tumour reactive T cells has gained significant therapeutic interest in tumour immunotherapy. Within this field, targeting CTAs has emerged as a particularly promising approach due to their tumour-restricted specificity (32). To broaden their clinical impact, it is critical to expand CTA-targeting immunotherapies, thereby increasing eligible patients and applicable cancer types.

The *SSX* gene family represents a group of highly homogeneous nuclear-localized proteins whose abnormal expression has been reported to be linked to advanced stages of various cancers (33). Among these genes, *SSX2* stands out as a promising candidate due to its tumour prevalence and immunogenicity (34). Pre-existing host immune recognition of *SSX2*-expressing cancerous cells further suggests that engineered T cells targeting this antigen may achieve enhanced persistence and efficacy, positioning *SSX2* as an attractive candidate for developing next-generation ACT or TCR-based therapeutic approaches (18, 26). A recent successful attempt has been made in generating HLA-A*02:01/SSX2_41–49_ epitope reactive TCR-mimic CAR-T cells against AML (35), highlighting the importance of identifying naturally occurring, functionally competent SSX2-reactive TCRs that could potentially benefit larger cohorts of cancer patients.

To this end, we systematically characterised HLA-A*02:01/SSX2_41-49_-specific TCRs previously identified from cancer patients (26). To ensure precise genetic modification and avoid random gene integration caused by lentivirus (LV) transduction, we adopted a virus-free orthotopic TCR replacement system for T cell modification (27). This approach enables precise gene integration and places transgenic TCR expression under physiological control. It also prevents TCR mispairing and provides a consistent platform for TCR functional evaluation (28). Our study identified a naturally occurring, SSX2-reactive TCR with high functional avidity. This TCR showed superior tumour suppression potential and did not show increased expression of the exhaustion markers examined under these conditions, highlighting its translational potential for future clinical applications (36, 37).

While CD8^+^ T cells are key mediators of direct tumour killing, their durable tumour control is often limited by their functional exhaustion and poor persistence (38). Incorporating CD4^+^ T cells offers synergistic effects to enhance overall anti-tumour immunity (39, 40). However, the TME frequently lacks predefined MHC-II restricted tumour epitopes to naturally elicit such responses. TCR-engineered T cells thus offer a promising strategy to redirect CD4^+^ T cells toward otherwise non-responsive tumour antigens (40, 41). Here, we showed the high functional avidity TCR enabled potent CD4^+^ T cell responses towards the MHC-I-restricted SSX2_41–49_ epitope, whereas the low functional avidity TCR showed minimal responses under the same stimulation. Upon activation, these reprogrammed CD4^+^ T cells deliver critical “helper” functions via the release of multiple cytokines and contribute directly to cytotoxic tumour control. Consequently, the unique ability of this high functionality TCR in harnessing both CD4^+^ and CD8^+^ T cells for synergistic anti-tumour immunity positions it as a highly promising candidate for TCR-based therapeutics.

Beyond conventional αβ T cells, we also proved the feasibility and effectiveness of redirecting alternative unconventional T cells for ACT, an approach that represents a promising strategy to broaden therapeutic applications (42). These innate-like lymphocytes bridge innate and adaptive immunity, offering rapid effector functions, tissue residency and potent tumour cytotoxicity (23, 43). However, their translational potential has been restricted by their low abundance and incomplete characterization (21). Engineering them with tumour-specific TCRs can precisely redirect and enhance their natural anti-tumour capacity. Here, our “proof-of-concept” framework shows that engineering these T cells with tumour-specific TCRs integrates their innate anti-tumour potential with precise antigen recognition. Moreover, their MHC-independent nature creates opportunities for generating “off-the-shelf” products with robust anti-tumour immune responses (44).

Adoptive transfer of affinity-enhanced MAGE-A3 targeting TCR-T cells has been reported to cause lethal cross-reactivity with the cardiac antigen titin (45), underscoring the importance of rigorous safety evaluation before clinical translation. In the context of the HLA-A*02:01-restricted SSX2_41-49_-specific TCR investigated in this study, Abate-Daga et al. previously assessed homologous peptides from SSX family members and selected non-SSX human proteins, showing substantially stronger recognition of SSX2_41–49_ than of the tested homologous peptides (19). To extend this assessment, we performed a preliminary *in silico* screen of the human proteome and identified a limited set of candidate self-peptides with sequence similarity to the KASEKIFYV peptide and predicted HLA-A*02:01 binding and presentation. However, these analyses do not fully replace comprehensive TCR-specific safety testing. Further studies should incorporate experimentally defined peptide-recognition motifs, *in silico* prediction tools (46), and intensive *ex vivo* safety screening strategies, including normal tissue screening and yeast/phage display platforms (47, 48). Another limitation of this study is the lack of a comprehensive *in vivo* evaluation of the adoptively transferred T cell dynamics post-reinfusion. While *in vitro* assays only capture the direct TCR-tumour interactions, *in vivo* tumour xenograft animal models are essential to evaluate T cell behaviour in the context of the complex TME. Such approaches would ultimately provide critical insights into a selected TCR prior to clinical applications.

In summary, this study identified a CD8-independent, SSX2-reactive high functional avidity TCR capable of redirecting both conventional and unconventional T cells for effective tumour control. Collectively, this work establishes a framework for developing next-generation tumour immunotherapy by leveraging a CTA-targeting and functionally competent TCR. The development of more broadly applicable TCR-based therapeutic strategies would ultimately benefit a broad spectrum of cancer patients in future clinical translation.

## Data Availability

The raw data supporting the conclusions of this article will be made available by the authors, without undue reservation.
