# Supplementary material for: Patient-derived high functional avidity TCR empowers diverse T cell subsets for tumour immunotherapy
Source: Front Immunol. 2026 Oct 19;17:1964649. doi: 10.3389/fimmu.2026.1964649 (PMC13724408; doi:10.3389/fimmu.2026.1964649)
Supplement: Supplementary file 1 [file DataSheet1.docx]

**Supplementary figures**

**Supplementary Figure 1. Functional evaluation of SSX2-reactive TCRs using TCR-transduced Jurkat cells.** Flow cytometry analysis of CD69 expression on the surface of SSX2-reactive TCR-transduced Jurkat cells after co-culture with SSX2_41-49_ peptide titrated HLA-A*02:01 positive B cells. Peptide concentration: μM.

**Supplementary Figure 2. HLA-A*02:01-restricted recognition by SSX2-reactive TCR-T cells.** (A) Flow cytometry analysis of HLA-A*02:01 expression in parental JEG-3 and HLA-A*02:01 transduced JEG-3 (A2^+^ JEG-3) cell lines. (B) Representative intracellular cytokine staining plots showing IFN-γ and TNF-α production by SSX2-reactive TCR-T cells after co-culture with parental JEG-3 or HLA-A*02:01-expressing JEG-3 cells pulsed with increasing concentrations of SSX2_41-49_ peptide.

**Supplementary Figure 3. HLA-A*02:01^+^ SSX2-expressing cell line identification.** HLA-A*02:01 staining and relative *SSX2* mRNA expression of different cell lines (A) Flow cytometry analysis of HLA-A*02:01 expression on the cell surface. (B) qPCR amplification of *SSX2* mRNA expression of different cell lines. *SSX2* mRNA fold change in each cell line was normalized to the *SSX2* mRNA expression of HCT 116 cancer cell line (n=3).

**Supplementary Figure 4. Generation of SSX2-overexpressing cancer cell lines.** (A) Schematic overview of GFP empty vector (EV) and SSX2-GFP lentiviral vector design. (B) Representative flow cytometry analysis of GFP expression by tumour cells after transduction with either the GFP empty vector or the SSX2-GFP lentiviral vector.

**Supplementary Figure 5. Exhaustion marker expression of TCR-T cells.** (A, B) Flow cytometry analysis of exhaustion marker expression on CD8^+^ TCR-T cell surface 72h after co-culture with SSX2-expressing HCT 116 (A) and MESO 174 (B) tumour cell lines. Data are presented as mean ± SD. Unpaired Student’s *t*-tests (A, B) were performed, and two-tailed P values were calculated for comparison. *P < 0.05; **P < 0.01; ***P < 0.001, n.s., not significant.

**Supplementary Figure 6. *SSX2* gene expression analysis across different cancers.** Analysis of *SSX2* gene expression (FPKM) in tumour, adjacent paratumour and healthy tissues based upon data generated by the TCGA Research Network ([https://www.cancer.gov/tcga](https://www.cancer.gov/ccg/research/genome-sequencing/tcga)). LUAD, lung adenocarcinoma; LUSC, lung squamous cell carcinoma; LIHC, liver hepatocellular carcinoma; BRCA, breast invasive carcinoma; PRAD, prostate adenocarcinoma; BLCA, bladder urothelial carcinoma; STAD, stomach adenocarcinoma; COAD, colon adenocarcinoma; UCEC, uterine corpus endometrial carcinoma; UCS, uterine carcinosarcoma.

**Supplementary Figure 7. CD4^+^ TCR-T cell responses towards vaccinia virus infected B cell lines.** Representative flow cytometry plots showing intracellular IFN-γ and TNF-α release by CD4^+^ TCR-T cells following co-culture with SSX2- or SSX4-expressing vaccinia virus infected B cell lines (BCLs) or peptide-pulsed controls. (A) TCR-T cell responses towards HLA-A*02:01 positive BCLs. (B) TCR-T cell responses towards HLA-A*02:01 negative BCLs.

**Supplementary Figure 8. Killing capacity of TCR redirected CD4^+^ T cells.** (A) Flow cytometry analysis of killing capacity of SSX2-reactive CD4^+^ TCR-T cells towards SSX2_41-49_ peptide-pulsed T2 cells at 1 ng/mL, 10 ng/mL, 100 ng/mL or 1000 ng/mL peptide concentrations. (B) Killing index of SSX2-reactive CD4^+^ TCR-T cells towards SSX2_41-49_ peptide-pulsed T2 cells at 10 ng/mL and 100 ng/mL peptide concentrations at different time points. Data are presented as mean ± SD. Unpaired Student’s *t*-test (A) and paired Student’s *t*-test (B) were performed, two-tailed P values were calculated for comparison. *P < 0.05; **P < 0.01; ***P < 0.001, n.s., not significant.

**Supplementary Figure 9. Unconventional T cell sorting strategy.** Gating strategies to identify different subsets of unconventional T cells from PBMCs. (A) PBMCs were gated on lymphocytes, single cells and live cells. (B) Individual markers and the gating strategy for different subsets of unconventional T cells. NKT, MAIT and γδ T cells were identified as CD3^+^CD56^+^, CD161^+^Vα7.2^+^ and γδ TCR^+^ populations, respectively.

**Supplementary Figure 10. Orthotopic TCR replacement strategy in γδ T cells.** Schematic overview of generating HDR γδ TCR-T cells at the endogenous *TRDC* gene locus for SSX2-reactive TCR replacement. DNA donor template was designed to carry the exogenous murinized SSX2 TCR sequence and the flanking regions of the *TRDC* gene.

**Supplementary Figure 11. Functional evaluation of SSX2 TCR-redirected T cells towards SSX2-expressing tumour cell line.** Representative flow cytometry plots showing intracellular IFN-γ and TNF-α release after SSX2-reactive conventional αβ TCR-T cells and unconventional TCR-T cells co-cultured with SSX2-transduced HCT 116 cells.
